# The interplay of cognitive and motor impairment in the occurrence of dementia and presence of brain pathologies: a community‐based cohort study

**DOI:** 10.1002/alz.084892

**Published:** 2025-01-03

**Authors:** Jiao Wang, Anna‐Karin Welmer, David A. Bennett, Weili Xu

**Affiliations:** ^1^ Army Medical University, Chongqing, Chongqing China; ^2^ Aging Research Center, Karolinska Institutet, Stockholm Sweden; ^3^ Rush University, Chicago, IL USA

## Abstract

**Background:**

Evidence on the additive effect of mild cognitive impairment (MCI) and motor function (MF) impairment on dementia and brain pathologies is sparse. We aimed to explore the incidence of dementia including Alzheimer’s dementia, and brain pathologies among people with both MCI and low MF.

**Methods:**

Within the Rush Memory and Aging Project, 1,814 dementia‐free participants (mean age: 79.7±7.4 years; 74.4% female) were identified at baseline and followed up to 24 years to detect incident dementia. Baseline motor function was assessed by a composite score of dexterity, gait, and hand strength, and tertiled (low, moderate, and high). MCI was defined as the presence of cognitive impairment but not meeting criteria for dementia. Then, four groups were classified: (I) normal, (II) isolated low MF, (III) isolated MCI, and (IV) MCI & low MF. Dementia including Alzheimer’s dementia was diagnosed following international criteria. During the follow‐up, 850 participants died and underwent autopsies for neuropathological assessment (including Alzheimer’s disease, vascular, and other brain pathologies). Data were analyzed using Cox regression and logistic regression.

**Results:**

At baseline, 964, 380, 245, and 225 participants were in the above four groups, respectively. During the follow‐up (median [interquartile range]: 6 [3‐9] years), 506 (27.89%) participants developed dementia, including 469 (25.85%) Alzheimer’s dementia. Compared to the normal group, the multivariable‐adjusted hazard ratios (95% confidence intervals) of MCI & low MF was 5.65 (4.26‐7.50) for dementia and 5.78 (4.31‐7.76) for Alzheimer’s dementia. A significant additive interaction was found between MCI and low MF on dementia risk (AP 0.49: 0.31‐0.66). In the brain pathological data analysis, MCI & low MF was significantly related to higher neurofibrillary tangles (odds ratio 1.99: 1.25‐3.16), cerebral atherosclerosis (2.40: 1.39‐4.12), Lewy bodies (1.71: 1.01‐2.90), and hippocampal sclerosis (3.50: 1.56‐7.86), compared to the normal group.

**Conclusion:**

Adding MF to the assessment of MCI could better detect dementia risk. Both neurodegenerative and vascular mechanisms may underlie the interplay between MF and MCI.

